# Minimally Invasive Transforaminal Lumbar Interbody Fusion with Unilateral Pedicle Screw Fixation: Comparison between Primary and Revision Surgery

**DOI:** 10.1155/2014/919248

**Published:** 2014-05-14

**Authors:** Moo Sung Kang, Jeong Yoon Park, Kyung Hyun Kim, Sung Uk Kuh, Dong Kyu Chin, Keun Su Kim, Yong Eun Cho

**Affiliations:** Department of Neurosurgery, Gangnam Severance Hospital, Spine and Spinal Cord Institute, Yonsei University College of Medicine 712, Eonju Ro Gangnam-gu, Seoul 135-720, Republic of Korea

## Abstract

Minimally invasive surgery with a transforaminal lumbar interbody fusion (MIS TLIF) is an important minimally invasive fusion technique for the lumbar spine. Lumbar spine reoperation is challenging and is thought to have greater complication risks. The purpose of this study was to compare MIS TLIF with unilateral screw fixation perioperative results between primary and revision surgeries. This was a prospective study that included 46 patients who underwent MIS TLIF with unilateral pedicle screw. The patients were divided into two groups, primary and revision MIS TLIF, to compare perioperative results and complications. The two groups were similar in age, sex, and level of operation, and were not significantly different in the length of follow-up or clinical results. Although dural tears were more common with the revision group (primary 1; revision 4), operation time, blood loss, total perioperative complication, and fusion rates were not significantly different between the two groups. Both groups showed substantial improvements in VAS and ODI scores one year after surgical treatment. Revision MIS TLIF performed by an experienced surgeon does not necessarily increase the risk of perioperative complication compared with primary surgery. MIS TLIF with unilateral pedicle screw fixation is a valuable option for revision lumbar surgery.

## 1. Introduction

Transforaminal lumbar interbody fusion (TLIF) has become a popular and well-established technique after being introduced by Harms and Rolinger in 1982 [1]. Recently, advances in minimally invasive surgical techniques have allowed minimally invasive transforaminal lumbar interbody fusion (MIS TLIF) to reduce the complications associated with open surgical techniques, while simultaneously demonstrating similar clinical results [2–6].

Generally, bilateral pedicle screw fixation after MIS TLIF is accepted as a standard procedure for treating symptomatic spinal pathologies, such as degenerative spinal stenosis, spondylolisthesis, recurrent disc herniation, postlaminectomy instability, and deformity [7–9]. Some authors have recently demonstrated that unilateral pedicle screw fixation is as effective for spinal fusion as bilateral screw fixation after MIS TLIF, and spinal stenosis without instability can be a good indication for MIS TLIF with unilateral pedicle screw fixation [10–12].

Reoperation in the lumbar spine is challenging and is thought to have a greater risk for complications than primary surgery. Several studies recently reported that conventional TLIF by revision surgery did not increase the risk of perioperative complications compared with primary surgery, including dural tear and neurological injuries, and Selznick et al. reported that MIS TLIF as revision surgery is as safe as conventional TLIF [9, 13, 14]. However, to the best of the authors' knowledge, no current studies have directly compared the effects of previous lumbar surgery on the complication rates of MIS TLIF with primary MIS TLIF. The purpose of this study was to compare the perioperative results of MIS TLIF with unilateral screw fixation between primary and revision surgery.

## 2. Materials and Methods

### 2.1. Patient Population

Forty-six consecutive patients that had undergone MIS TLIF with unilateral percutaneous pedicle screw fixation by tubular retractor system from January 2010 to January 2012, either as a primary surgery or a revision, were prospectively enrolled in this study. Only one segment was used as an indication for surgical treatment (L4/5 or L5/S1), in addition to degenerative disc disease without instability including spinal stenosis, either primary or revision. We excluded patients treated for nondegenerative purposes such as trauma, tumor, or infection, as well as patients with spondylolisthesis, stenosis with instability and those who had undergone previous fusion surgery. Patients were divided into primary (25 patients) and revision groups (21 patients). All revision groups underwent microscopic hemilaminectomy with discectomy previously, and MIS TLIF was done at previously operated side. All operations in this study were performed by the same surgeon (JYP). This project was approved by the Institutional Review Board of Gangnam Severance Hospital, Yonsei University College of Medicine, and the authors acquired patient consent for trial participation.

### 2.2. Operative Techniques [6]

**Figure 1:**
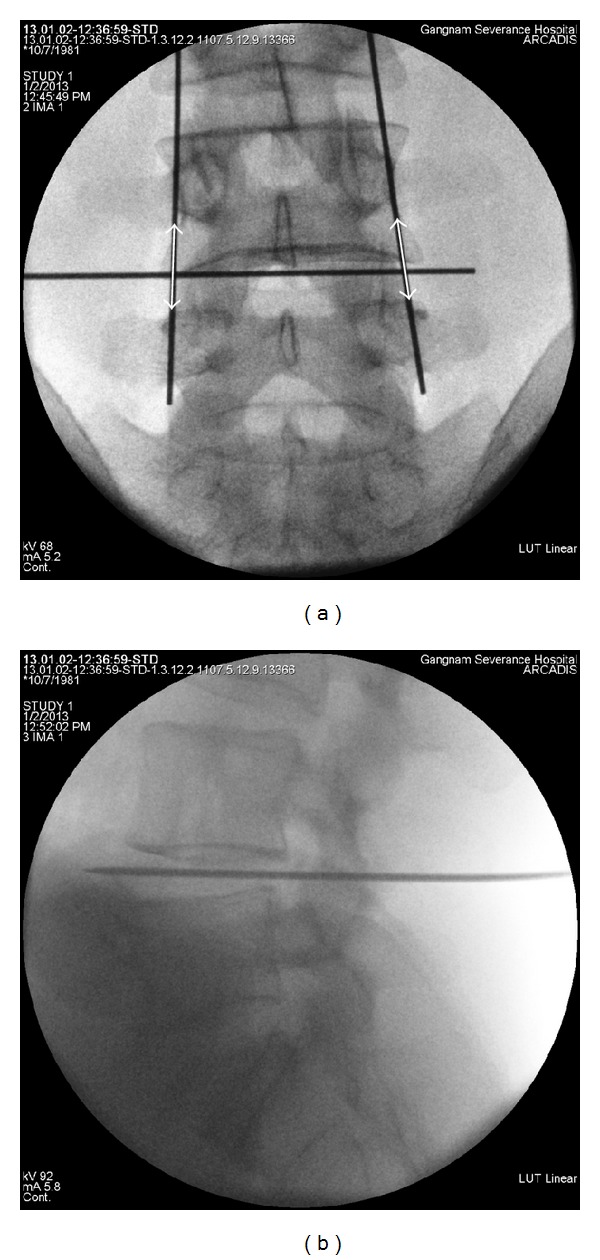
Fluoroscopic guidance in the AP view shows a mark on the skin at the disc space and the lateral pedicle line. A vertical skin incision was made at the disc space 15 mm cranially and 10 mm caudally (white arrow) (a). Lateral view fluoroscopy was used to confirm the lateral disc space (b).

The MIS TLIF procedure was performed on the more symptomatic side under general anesthesia with a C-arm image intensifier entry point. C-arm guidance was used to determine the operative level and mark the line in the fluoroscopic AP view (Figure 1(a)) and lateral view (Figure 1(b)) for tubular retractor system insertion. After a vertical skin incision (length: 25 mm) (Figure 1(a)), a tubular retractor (diameter: 22 mm, MetRx; Medtronic Sofamor Danek, Memphis, TN, USA) was introduced to the facet under fluoroscopic guidance. Monopolar cautery and pituitary forceps were used to expose the facet complex, and the total facetectomy was performed with a high-speed drill and osteotome. After a complete facetectomy, the ligamentum flavum was removed to expose the lateral border of the ipsilateral nerve root. The tubular retractor was angled medially, and the patient was tilted laterally to decompress the contralateral side. Next, extensive decompression was performed, which included decompression of the central stenosis and the contralateral side.

**Figure 2:**
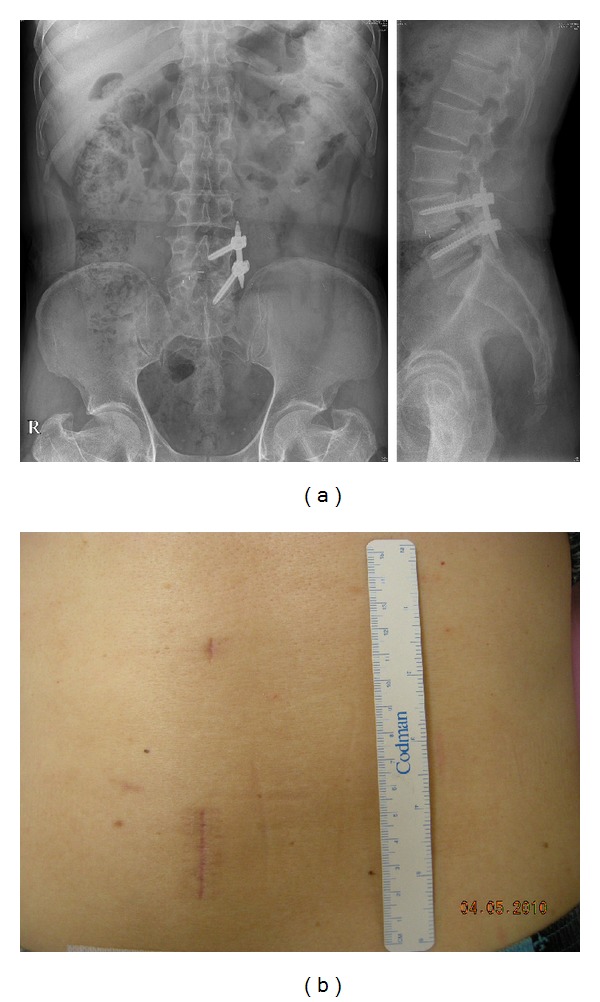
Minimally invasive transforaminal lumbar interbody fusion with unilateral percutaneous pedicle screw fixation. X-ray (a) and final skin incision (b). An ipsilateral incision for the tubular retractor, an ipsilateral percutaneous pedicle screw system, and an upper incision for rod insertion (b).

A discectomy was performed, and a single long polyether-etherketone (PEEK) interbody bullet-shaped cage (Capstone; Medtronic Sofamor Danek, Memphis, TN, USA) filled with only autologous local bone was inserted. Posterolateral fusion was not performed due to the small surgical field of the tubular retractor (diameter 22 mm). After interbody fusion, the tubular retractor was removed, and ipsilateral percutaneous pedicle screw system (Sextant; Medtronic Sofamor Danek, Memphis, TN) was inserted through the same trajectory (Figures 2(a) and 2(b)).

### 2.3. Perioperative Results and Clinical Outcome Measures

The study assessed the perioperative results related to the operative procedure such as blood loss, operation time, and perioperative complications. The perioperative complication was defined as any adverse event that occurred intraoperatively or within six weeks of the MIS TLIF [14]. The complications included dural tear, pedicle screw, or cage malposition, cage migration, new or increased neurologic deficit, blood vessel damage, deep venous thrombosis, pulmonary embolism, infection, cerebrospinal fluid (CSF) leakage, hematoma, and anemia or other complications that required patient readmission to the hospital.

The clinical outcomes were assessed with the Visual Analogue Scale (VAS) of leg and back pain and the Oswestry Disability Index (ODI) prior to surgery and at three months, six months, and one year after surgery. Radiologic outcomes about fusion were determined by an independent neurosurgeon and a neuroradiologist, who were blinded to the treatment details. Fusion rates were assessed with the Bridwell grading system, and CT and radiographic findings were assessed 1 year after surgical treatment [15].

All analyses were performed with SPSS version 15.0 (SPSS, Inc., Chicago, IL). Demographic data and complication rates of primary and revision groups were assessed by Fisher's exact test, and the perioperative results and clinical data were assessed by the Mann-Whitney Test. *P* values <0.05 were considered statistically significant.

## 3. Results

Forty-six patients that had undergone MIS TLIF with unilateral pedicle screw fixation (27 men and 19 women) were identified. The mean follow-up period was 17.4 months, and the mean age was 54.7 years. Of the 46 patients, 25 (primary group) had no history of lumbar surgery and 21 (revision group) had undergone previous microscopic hemilaminectomy with discectomy. All demographic data and perioperative results are shown in Table 1. Comparison between the two groups did not show any significant differences with respect to the number of patients, age (primary 57.4; revision 51.5 years), gender (male/female: primary 14/11; revision 13/8), follow-up period (primary 17.6; revision 16.3 months), or level of fusion (L4-5/L5-S1: primary 18/7; revision 14/7) (Table 1).

Perioperative results (operation time: 88.8 versus 88.4 minutes and blood loss: 94.4 versus 87.5 mL) were not significantly different between the two groups. The only complications were dural tear and cage migration. Although there was no statistical difference, the dural tear rate was higher in the revision group (primary 1; revision 4, *P* = 0.16), and the numbers of complications (primary 3; revision 4, *P* = 0.44) were not significantly different between the two groups (Table 1). The primary group contained one patient that underwent revision surgery due to cage migration.

**Figure 3:**
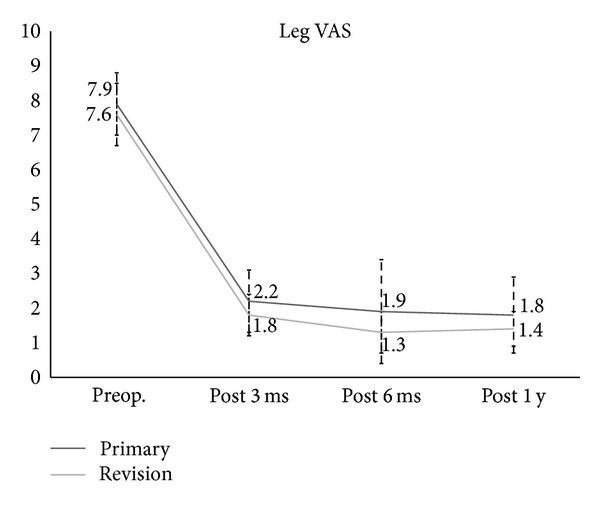
The Visual Analogue Scale (VAS) of leg pain for the primary group and the revision group.

**Figure 4:**
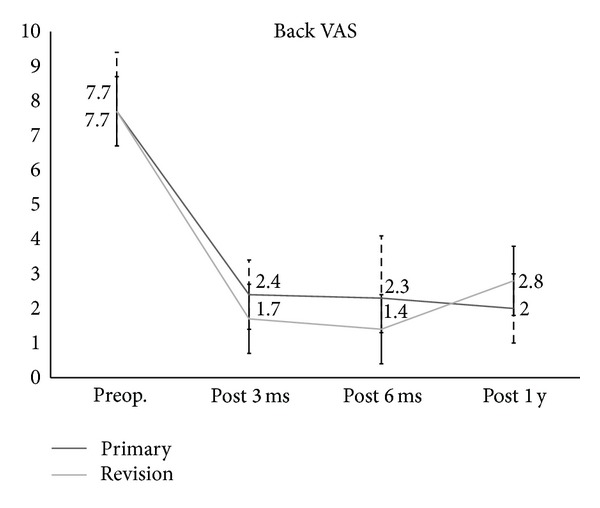
The Visual Analogue Scale (VAS) of back pain for the primary group and the revision group.

**Figure 5:**
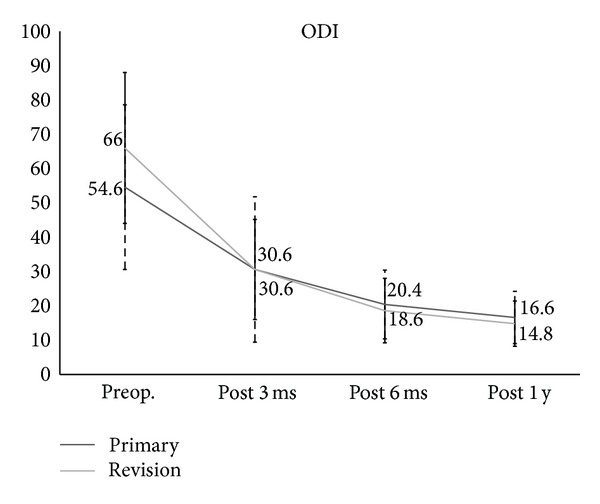
The Oswestry Disability Index scores for the primary group and the revision group.

In regard to clinical outcomes, the VAS for leg pain (primary: 7.9 to 1.8, revision: 7.6 to 1.4) and back pain (primary: 7.7 to 2, revision: 7.7 to 2.8) were significantly improved at one year after the operation (Figures 3 and 4). The ODI score also significantly improved at one year after the operation (primary: 54.6 to 16.6, revision 66 to 14.8) (Figure 5), but there were no significant differences in the clinical results between the two groups. In radiologic outcomes according to the Bridwell grading system, fusion grades in primary group were grade I in 80% (*n* = 21), grade II in 20% (*n* = 5); in revision group, fusion grades were grade I in 76.2% (*n* = 16), grade II in 23.8% (*n* = 5). Since fusion is defined as grade I, primary group had a fusion rate of 80% (*n* = 20) and revision group had a fusion rate of 76.2% (*n* = 16), and there were no significant differences between groups (*P* = 1.00; Table 1).

## 4. Discussion

Unilateral or bilateral pedicle screw insertion after MIS TLIF is still controversial. Goel et al. first reported better perioperative outcomes (short operation time, less blood loss, and short hospital stay) of unilateral pedicle screw fixation than bilateral pedicle screw fixation with posterolateral fusion, and additional clinical trials have reported that unilateral pedicle screw fixation is as effective as bilateral pedicle screw fixation in fusion rate and long term clinical results [16–19]. Bilateral pedicle screw fixation after MIS TLIF is generally accepted as a standard procedure for symptomatic spinal pathology treatment, [7–9] but many clinical trials have reported positive outcomes (short operation time, less blood loss, and short hospital stay) associated with unilateral pedicle screw fixation after conventional TLIF and MIS TLIF [10–12]. Although previous reports showed that unilateral pedicle screw fixation after MIS TLIF is comparable to bilateral screw fixation, we already reported that less fusion rate (unilateral 84.6%; bilateral 96.3%) and more postoperative scoliosis change rate (unilateral 23.1%; bilateral 3.7%) with unilateral screw fixation than bilateral screw fixation after MIS TLIF [20]. The authors reported in a previous study that numerous degenerative disc diseases are associated with instability, which supports the report that bilateral screw fixation has better outcomes than unilateral screw fixation [20]. Because MIS TLIF requires complete unilateral facet joint removal and can result in iatrogenic instability, bilateral screw fixation may prove to be more beneficial than unilateral screw fixation in patients with preoperative instability [5, 6, 21, 22]. In present study, to avoid the inferiority of unilateral screw fixation after MIS TLIF, the author only did MIS TLIF with unilateral pedicle screw fixation at the patient who did not have preoperative instability either primary or revision, and there were no complications related with unilateral percutaneous pedicle screw fixation such as postoperative scoliotic change [20]. Ultimately, MIS TLIF with unilateral pedicle screw fixation may prove to be a good option for degenerative lumbar disc disease that is not associated with instability, because the number of metal implants is small (Figure 2(a)), insertion wound is very small (Figure 2(b)), operation time is shorter, blood loss is little, and fusion rate is acceptable (76~80%) (Table 1) [10, 20].

Lumbar fusion surgery has been used as a salvage procedure for patients who have previously undergone lumbar surgical procedures [14]. However, revision spine surgery is challenging and has been reported to pose a greater risk for complications. The most common complication of conventional revision lumbar surgery is an incidental dural tear, and the incidence of dural tear varies from 8 to 21% [9, 14, 23, 24]. Currently, only few studies have discussed conventional and MIS TLIF by revision surgery. Recently, Tormenti et al. reported that conventional TLIF by revision surgery was more frequently associated with dural tear than primary TLIF (18 versus 11%), although wound infection (3.7 versus 3.8%) and other complications (screw misplacement, cage migration, and retroperitoneal injury) were not significantly different between revision and primary TLIF [25]. The results of the previous study about revision conventional TLIF had comparable dural tear rates to the former conventional revision fusion technique except TLIF [2, 26] and have proposed that conventional TLIF may prove to be a good option for revision surgery [25]. Khan et al. also reported that conventional TLIF, as a revision surgery, was not associated with greater perioperative complication rates than primary conventional TLIF, and revision conventional TLIF did not increase the risk of complication compared with primary TLIF [14].

As a revision surgery, Wang et al. reported that MIS TLIF showed less blood loss, postoperative back pain, infection, and less dural tears (12 versus 18%) than conventional TLIF; additionally, even though the radiation time was longer in MIS TLIF, they reported that MIS TLIF is a safe and effective treatment for revision lumbar surgery [27]. Selznick et al. also suggested that, though MIS TLIF for revision surgery was associated with more frequent dural tears than primary surgery (29 versus 4%) when performed by an experienced surgeon, it may be a valuable option for revision lumbar surgery [9]. However, previous studies have not prospectively and directly compared the effects of previous lumbar surgeries on the complication rates of revision MIS TLIF with primary MIS TLIF. MIS TLIF itself needs time to overcome learning curve, and from previous studies, we already know that it needs more than 20 cases experience in MIS TLIF to overcome learning curve [26, 28]. The authors started MIS TLIF from 2008 and previous results of MIS TLIF as primary surgery by us were already reported [6, 20] and experienced more than 100 cases MIS TLIF before starting this study, so we are sure that learning curve did not make any problems at this study.

**Figure 6:**
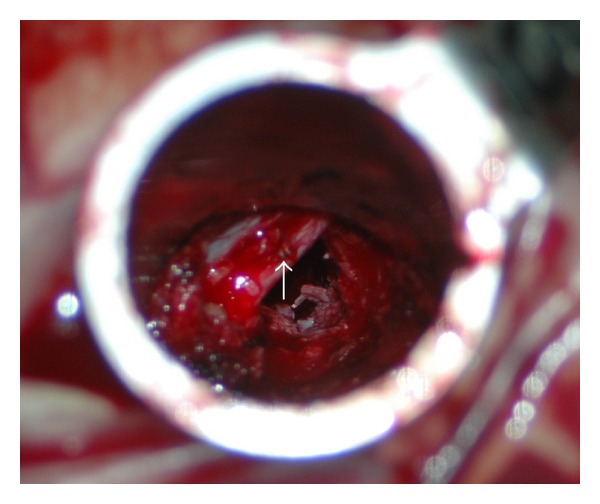
Operation image during minimally invasive transforaminal lumbar interbody fusion (MIS TLIF). Dural tear was directly repaired with a small vascular clip (white arrow).

We determined that the rate of complications associated with MIS TLIF with unilateral pedicle screw fixation was not statistically significant between primary (12%) and revision (19%) (Table 1). Dural tear (5 cases, 11%) was the most frequent postoperative complication during MIS TLIF (primary 1, 4%; revision 4, 19%), and this rate is similar to previous revision surgery studies [9, 14, 23, 24]. Among the five cases of dural tear, two patients underwent direct repair with vascular clip (Figure 6), and three patients did not require direct repair because of the small size of the tear; additionally, there were no patients with CSF leakage that required an additional hospital stay. There were no other perioperative complications during revision MIS TLIF. Although dural tear was more common in the revision group, the operation time, blood loss, and clinical results were not significantly different between primary and revision MIS TLIF with unilateral pedicle screw fixation.

There were limitations to this study. A small number of cases were included in each group, patients were not randomly assigned into each group, and follow-up period was relatively short. However, this is the first prospective study of revision MIS TLIF with unilateral pedicle screw fixation. Future studies that include a long-term follow-up with a large number of patient cases should be conducted.

## 5. Conclusions

This study demonstrated that MIS TLIF with unilateral pedicle screw fixation as a revision surgery for degenerative lumbar disease without instability demonstrated similar perioperative results to primary surgery. MIS TLIF with unilateral pedicle screw fixation is a safe and effective alternative technique to spinal arthrodesis for revision lumbar surgery when used on the patients without instability and when performed by an experienced surgeon.

## Figures and Tables

**Table 1:** Characteristics of patients who underwent minimally invasive TLIF.

Characteristics	MIS TLIF		*P* value
	Primary (%)	Revision (%)	
Number of patients	25	21	
Mean age (yrs)	57.4 ± 14.1	51.5 ± 12.6	0.19*
Sex			0.77^†^
Male	14	13	
Female	11	8	
Follow-up (ms)	17.6 ± 4.7	16.3 ± 3.2	0.45*
Level of fusion			0.76^†^
L4-5	18	14	
L5–S1	7	7	
Operation time (mins)	88.8 ± 42.6	88.4 ± 20.7	0.66*
Blood loss (mL)	94.4 ± 122.1	87.5 ± 62.6	0.70*
Complication	3 (12)	4 (19)	0.44^†^
Dural tear	1 (4)	4 (19)	0.16^†^
Cage migration	2 (8)	0	1.00^†^
Others	0	0	—
Reoperation	1^‡^	0	1.00^†^
Fusion			
Grade I	20 (80)	16 (76)	1.00^†^
Grade II	5	5	

Between groups comparison with *Mann-Whitney test and ^†^Fisher's Exact test. ^‡^Reoperation was done due to cage migration.
